# Connexin expression and gap-junctional intercellular communication in ES cells and iPS cells

**DOI:** 10.3389/fphar.2013.00085

**Published:** 2013-07-03

**Authors:** Masahito Oyamada, Kumiko Takebe, Aya Endo, Sachiko Hara, Yumiko Oyamada

**Affiliations:** ^1^Department of Food Science and Human Nutrition, Faculty of Human Life Sciences, Fuji Women's UniversityIshikarishi, Japan; ^2^Department of Surgical Pathology, Tonan HospitalSapporo, Japan

**Keywords:** connexins, gap-junctional intercellular communication, ES cells, iPS cells, differentiation, reprogramming, pluripotency

## Abstract

Pluripotent stem cells, i.e., embryonic stem (ES) and induced pluripotent stem (iPS) cells, can indefinitely proliferate without commitment and differentiate into all cell lineages. ES cells are derived from the inner cell mass of the preimplantation blastocyst, whereas iPS cells are generated from somatic cells by overexpression of a few transcription factors. Many studies have demonstrated that mouse and human iPS cells are highly similar but not identical to their respective ES cell counterparts. The potential to generate basically any differentiated cell types from these cells offers the possibility to establish new models of mammalian development and to create new sources of cells for regenerative medicine. ES cells and iPS cells also provide useful models to study connexin expression and gap-junctional intercellular communication (GJIC) during cell differentiation and reprogramming. In 1996, we reported connexin expression and GJIC in mouse ES cells. Because a substantial number of papers on these subjects have been published since our report, this Mini Review summarizes currently available data on connexin expression and GJIC in ES cells and iPS cells during undifferentiated state, differentiation, and reprogramming.

## Introduction

Gap junctions are cell–cell communicating junctions that consist of multimeric proteins called connexins and mediate the exchange of low-molecular-weight metabolites and ions between contacting cells (Oyamada et al., 2013). Gap-junctional intercellular communication (GJIC) has long been hypothesized to play a crucial role in the maintenance of homeostasis, morphogenesis, cell differentiation, and growth control in multicellular organisms. Discoveries of human genetic disorders due to mutations in connexin genes and experimental data on connexin knockout mice provide direct evidence that gap junctional intercellular communication is essential for tissue functions and organ development and that its dysfunction causes diseases. Connexin-related signaling also involves extracellular signaling (hemichannels) and non-channel intracellular signaling.

GJIC during embryonal development has been demonstrated by using microelectrode impalements to monitor the cell-to-cell movement of ions (ionic coupling) and by microinjection of small-molecular-weight fluorescent dyes such as Lucifer yellow into a single cell and observation of the subsequent dye spread into the surrounding cells (dye coupling) (Lo and Gilula, 1979; Kalimi and Lo, 1988, 1989). It has been revealed that in many instances, GJIC is established within the first few cleavages and results in the entire embryo becoming interconnected as a syncytium. As development progresses, however, dye coupling delineates boundaries defining restrictions in GJIC that effectively segregate the developing embryo or tissue into a number of “communication compartment” domains. Thus, cells lying within a communication compartment are well coupled, exhibiting both ionic and dye coupling, whereas there is little or no coupling between cells situated across a compartment border. Such restriction of GJIC and the segregation of cells into communication compartment domains are almost always associated with embryogenesis and development.

Pluripotent stem cells, which include embryonic stem (ES) cells and induced pluripotent stem (iPS) cells, possess the ability to proliferate indefinitely without commitment *in vitro* and also differentiate into all cell lineages belonging to the three embryonic germ layers (Evans and Kaufman, 1981; Thomson et al., 1998; Takahashi and Yamanaka, 2006; Takahashi et al., 2007). ES cells are derived from the inner cell mass of the preimplantation blastocyst, whereas iPS cells are generated from many different types of somatic cells by overexpression of only a few pluripontency-related transcription factors. Many studies have demonstrated that mouse and human iPS cells are highly similar but not identical to their respective ES cell counterparts morphologically, functionally, and molecularly at the level of transcription and genome-wide distribution of chromatin modification. The potential to generate basically any differentiated cell types from ES cells and iPS cells offers the possibility to establish new models of mammalian development and to create new sources of cells for regenerative medicine (Robinton and Daley, 2012).

The *in vitro* differentiation system using ES cells and iPS cells also provides a useful model to study connexin expression and GJIC during the early stage of cell differentiation (Wong et al., 2008; Sharovskaya, 2011). In addition, the importance of understanding the regulation of connexin expression in differentiating pluripotent cells is recognized in regenerative medicine.

In 1996, we first reported the expression of connexin genes and GJIC during *in vitro* cardiomyocyte differentiation of mouse ES cells (Oyamada et al., 1996). Because a substantial number of papers on these subjects have been published since our first report, this Mini Review summarizes currently available data on connexin expression and GJIC in ES cells and iPS cells during undifferentiated state, differentiation, and reprogramming.

## Questions about connexin expression and gap-junctional intercellular communication in ES/iPS cells

Main questions about connexin expression and GJIC in ES/iPS cells that have been addressed thus far can be summarized as below:

1. What kinds of connexins are expressed in undifferentiated ES/iPS cells?
2. To what extent do undifferentiated ES/iPS cells communicate with each other via gap junctions?
3. What changes in connexin expression and GJIC occur during differentiation of ES/iPS cells?
4. What roles do connexin expression and/or GJIC play in maintenance of pluripotency in ES/iPS cells?
5. What changes in connexin expression and GJIC occur during induction of pluripotency in somatic cells (reprogramming)?
6. What roles do connexin expression and/or GJIC play in reprogramming?

## Currently available data on connexin expression and gap-junctional communication in ES cells

Table 1 summarizes results of published papers concerning connexin expression and GJIC in ES cells.

**Table 1:** **Connexin expression and GJIC in ES cells**.

**ES cell lines**	**Connexin expression in undifferentiated cells**	**GJIC in undifferentiated cells**	**Differentiation from ES cells**	**Connexin expression during differentiation**	**GJIC during differentiation**	**Methods used to determine the final phenotype of differentiated cells**	**References**
Mouse ES cells (J1)	Cx43^1^, Cx45^1^ Not detected: Cx40^1^	Present^3^	Cardiomyocytes	Cx40^1^, Cx43^1^, ^2^, Cx45^1^	Present^3^. Restricted to neighboring beating cells	Contraction, Ca^2+^-imaging, cardiac-specific gene expression	Oyamada et al., 1996
Mouse ES cells (D3)			Cardiomyocytes	Cx43^2^	Present^3^	Contraction, EM	Westfall et al., 1997
Mouse Cx43^−/−^ ES cells (R1)	Cx45^1^. No compensatory upregulation of Cx40^1^ and Cx45^1^	Very low GJIC^3^	Cardiomyocytes. Cx43 knockout did not significantly change either the time course, frequency of cardiomyocytic differentiation, or expression of cardiac-specific genes	Upregulation of Cx40^1^	Very low GJIC^3^	Contraction, cardiac-specific gene expression	Oyamada et al., 2000
Mouse ES cells (D3)	Cx43^1^, ^2^		Cardiomyocytes	Increases in Cx40^2^ and Cx43^2^ during cardiac differentiation		Contraction, cardiac-specific gene expression, electrophysiology	Van Kempen et al., 2003
Mouse ES cells (HM1)			Cardiomyocytes	Upregulation of Cx40^2^ at a peak around day 3 (hanging drop period) + 14		Cardiac-specific gene expression, ANEPPS fluorescence, electrophysiology	Fijnvandraat et al., 2003
Mouse ES cells (CCE)	Cx43^1^, ^2^, Cx45^1^, ^2^ No or very low expression: Cx37^1^, Cx40^1^		Cardiomyocytes (irregular contractions in Cx45^−/−^ cells)	Cx37^1^, Cx40^1^, Cx43^1^, ^2^, Cx45^1^		Contraction, Ca^2+^-imaging, multielectrode array, cardiac-specific gene expression, EM	Egashira et al., 2004
Human ES cells (H1, H7, H9, H14)	Cx43^1^, ^2^, Cx45^1^	Present^3^					Carpenter et al., 2004
Human ES cells (GE01, GE09, BG01, BG02, TE06)	Cx43^1^, Cx45^1^						Bhattacharya et al., 2004
Human ES cells (HES-3, HES-4)	Cx43^1^ (As one of the candidate human ES marker genes)						Richards et al., 2004
Human ES cells (HES-3, HES-4)	Cx43^1^, ^2^, Cx45^1^, ^2^	Present^3^					Wong et al., 2004, 2006
Mouse ES cells (Royan B1)			Cardiomyocytes	Presence of gap junctions in 21-day cardiomyocytes by EM		Cardiac-specific gene expression, EM, pharmacological reagents	Baharvand et al., 2005
Mouse ES cells (DBA/1LacJ)			Cardiomyocytes	Cx43^1^, Cx45^1^		Contraction, Ca^2+^-imaging, cardiac-specific gene expression, EM	Chaudhary et al., 2006
Human ES cells (BG01, H1)	Cx43^1^, ^2^, Cx40^1^, ^2^, Cx45^1^, ^2^, Cx25^1^, Cx26^1^, Cx30^1^, Cx30.2^1^, Cx30.3^1^, Cx31^1^, Cx31.1^1^, Cx31.9^1^, Cx32^1^, Cx36^1^, Cx37^1^, Cx46^1^, Cx47^1^, Cx59^1^, Cx62^1^ Not detected: Cx40.1^1^, Cx50^1^	Presence of GJIC^3^, ^5^ and hemichannels Extremely rare dye coupling between ES cells and feeder cells				Contraction, electrophysiology, cardiac-specific gene expression, EM, pharmacological reagents	Huettner et al., 2006
Cynomolgus monkey ES cells (CMK-6)	Cx43^1^		Embryoid bodies (EBs)	Suppression of Cx43 mRNA expression during EB differentiation			Yamamoto et al., 2007
Human ES cells (HES2, HES-3, ENVY)	Cx43^2^	Presence of GJIC mediated transport of shRNA					Wolvetang et al., 2007
Mouse ES cells (D3)	Cx43^1^, ^2^	Present^3^. Cx43 silencing inhibited GJIC, induced a loss of pluripotent state, and decreased in the proliferation rate	EBs GJIC blockers and Cx43-siRNA inhibited the formation of EBs from ES cells				Todorova et al., 2008
Mouse ES cells (HM1)	Cx43^1^, ^2^, Cx45^1^, ^2^, Cx31^1^, ^2^, Cx26^1^, Cx30.3^1^, Cx32^1^, Cx37^1^ Not detected: Cx26^2^, Cx29^1^, Cx30^1^, Cx30.2^1^, Cx31.1^1^, Cx32^2^, Cx33^1^, Cx36^1^, Cx37^2^, Cx40^1^, Cx46^1^, Cx47^1^, Cx50^1^, Cx57^1^	Present^3^, ^4^ Reduction of GJIC by decreased expression of Cx31 or Cx45 via RNA interference in Cx43^−/−^ ES cells did not lead to apoptosis					Worsdorfer et al., 2008
Mouse ES cells (D3): Sox1-promoter-GFP + ES cells and Cx43^−/−^ ES cells	Cx43^1^		Neuroectodermal cells Cx43^−/−^ ES cells showed a failure of oligodendrocyte development and an amplification of astrocytic cells	Wild-type ES cells showed “two-tailed” Cx43 expression with a maximum at day 7		Sox1-promoter-GFP, neuronal lineage-specific gene expression	Parekkadan et al., 2008
Human ES cells (hESM01)		Present^3^	Not characterized		Present but attenuated^3^ Restricted to differentiated cells Absence of GJIC between pluripotent and differentiating cells		Sharovskaya et al., 2009
Mouse ES cells (R1)			Cardiomyocytes	Cx43^2^ expression in cell sheets of mouse ES cell-derived cardiomyocytes		Cardiac-specific gene expression, multielectrode array	Matsuura et al., 2011

1 mRNA level;

2 Protein level;

3 dye coupling (Lucifer yellow etc.);

4 neurobiotin tracer coupling;

5 electrical coupling; EM, electron microscopy.

## Connexin expression and gap-junctional intercellular communication in iPS cells

Table 2 summarizes results of published papers concerning connexin expression and GJIC in iPS cells.

**Table 2:** **Connexin expression and GJIC in iPS cells**.

**iPS cell lines**	**Connexin expression in undifferentiated cells**	**GJIC in undifferentiated cells**	**Differentiation from iPS cells**	**Connexin expression during differentiation**	**GJIC during differentiation**	**Methods used to determine the final phenotype of differentiated cells**	**References**
Mouse iPS cells (O9), Mouse ES cells (E14.1)			Cardiomyocytes differentiated from iPS and ES cells with the use of a standard EB-based protocol	Cx43 in iPS cell- and ES cell-derived cardiomyocytes on day 22		Contraction, cardiac-specific gene expression, Ca^2+^-imaging, multielectrode array	Mauritz et al., 2008
Human iPS cells reprogrammed from primary keratinocytes	Cx43^2^						Aasen et al., 2008
Mouse iPS cells (O9, N10), Mouse ES cells (R1, D3)			Cardiomyocytes	Cx43 in iPS cell- and ES cell-derived cardiomyocytes		Contraction, cardiac-specific gene expression, multielectrode array, electrophysiology, pharmacological reagents	Pfannkuche et al., 2009
Mouse iPS cells reprogrammed without c-MYC			*In vivo* 3 germ layer differentiation, i.e., endoderm, ectoderm, and mesoderm. *In vitro* cardiomyocyte differentiation	Cx43 in iPS cell-derived cardiomyocytes *in vivo* and *in vitro*		Contraction, cardiac-specific gene expression, Ca^2+^-imaging, electrophysiology, EM	Martinez-Fernandez et al., 2009
Human iPS cells reprogrammed from HUVECs	Cx43^1^, Cx45^1^ Not detected: Cx37^1^	GJIC^3^ is re-established during reprogramming to pluripotency		HUVECs express Cx43^1^, Cx37^1^, and Cx45^1^	Low GJIC in HUVECs		Sharovskaya et al., 2012
Human iPS cells reprogrammed from human embryonic fibroblasts	Cx43^1^, ^2^, Cx25^1^, Cx26^1^, Cx30^1^, Cx30.2^1^, Cx30.3^1^, Cx31^1^, Cx31.1^1^, Cx31.9^1^, Cx37^1^, Cx40^1^, Cx45^1^, Cx46^1^, Cx47^1^, Cx59^1^, Cx62^1^. Cx43 increases during reprogramming	Present^3^		Human embryonic fibroblasts express Cx43^1^, ^2^ at low levels			Ke et al., 2013
Human iPS cell line (iMR90) Human ES cell lines (H7, RuES-2)			Cardiomyocytes differentiated from iPS and ES cells using a long-term (over 150 days) culture protocol	Significant increase in Cx43^1^, ^2^ expression in late-stage (80–120 days) cardiomyocytes vs. early stage (20–40 days) counterparts		Optical contraction analysis, electrophysiology, Ca^2+^-imaging, cardiac-specific gene expression, EM	Lundy et al., 2013

1 mRNA level;

2 protein level;

3 dye coupling (Lucifer yellow etc.); EM, electron microscopy.

Using human iPS cells, Sharovskaya et al. (2012) reported that GJIC is re-established during reprogramming to pluripotency: GJIC in incompletely reprogrammed cells was markedly decreased compared with that in the parental somatic cells, but GJIC in completely reprogrammed cells exceeded that in the parental somatic cells and was comparable to that in human ES cells. They drew an analogy between dramatic reduction of GJIC among the cells undergoing early reprogramming and weak GJIC or lack thereof among epithelial stem cells, such as keratinocyte stem cells, breast epithelial, and neural-glial stem cells, suggesting that changes in GJIC during early reprogramming might be associated with mesenchymal-to-epithelial transition (MET). They also showed that the opposite process of cell differentiation from the pluripotent state leads to the disruption of GJIC between pluripotent and differentiated cell subsets. However, GJIC is subsequently re-established *de novo* within each differentiated cell type *in vitro*, forming communication compartments within a histotype. Human iPS cells they utilized were derived from human umbilical vein endothelial cells (HUVECs) by lentiviral transfection with four transcription factors: KLF4, OCT4, SOX2, and C-MYC. To evaluate changes in GJIC during late stages of reprogramming, incompletely reprogrammed endo-iPSC10 cells at passage 6 and completely reprogrammed cells of the same line at passage 26 were studied. Incompletely reprogrammed iPS cells were characterized by residual expression of endothelial-specific genes including Cx37 and reduced expression of pluripotency-related genes. In addition, they compared expression of connexins in HUVEC, endo-iPS-10, 12, and human ES cells and found that only Cx37 and Cx43 expression varied significantly in the examined cell types. In incompletely reprogrammed iPS cells, Cx37 and Cx43 were expressed at the level similar to HUVEC. In faithfully reprogrammed iPS cells, cells lacked characteristics of parental HUVEC Cx37 expression, whereas Cx43 expression increased three- to five-fold.

Ke et al. (2013) demonstrated that Cx43 is specifically and highly enriched in undifferentiated human iPS cell lines during and after the reprogramming process. They also showed that iPS cells display functional GJIC and that Cx43 expression is gradually upregulated (~4.5-fold increase) during the reprogramming process. They observed that the Cx43 protein level increased gradually along with the expression of the pluripotency marker NANOG. Because Cx43 has been identified as a downstream target of the key pluripotency transcription factors OCT4, SOX2 and NANOG (Boyer et al., 2005), Cx43 expression might be upregulated by the key factors during reprogramming. They also found that the ectopic expression of Cx43 enhances the reprogramming efficiency (~3-fold increase), whereas the knockdown of endogenous Cx43 expression by RNAi reduces the efficiency, possibly by affecting the MET process, as reported by changes in E-cadherin expression. In addition, they showed that pharmacological GJIC inhibitors, CBX, 18-a-GA and the Cx43 mimetic peptide GAP27, did not affect the efficiency of iPS cell generation, suggesting that the effect of Cx43 on the efficiency of iPS cell generation may be attributed to the Cx43 protein itself but not to the function of GJIC, i.e., through a GJIC-independent pathway.

Taken together, these results suggest that Cx43 may represent a pluripotency marker of iPS cells and may play an important role in the reprogramming process.

Lundy et al. (2013) recently have developed a cell culture protocol capable of generating and maintaining highly purified human ES cell- and iPS cell-derived cardiomyocytes for several months *in vitro*. They have shown that these human ES cell- and iPS cell-derived cardiomyocytes are capable of maturing to a phenotype that more closely resembles adult cardiomyocytes in both structure and function. A robust induction of key cardiac structural markers including Cx43 has been demonstrated in late-stage ES cell- and iPS cell-derived cardiomyocytes. These findings suggest that ES cell- and iPS cell-derived cardiomyocytes are capable of slowly maturing to more closely resemble the phenotype of adult cardiomyocytes and may eventually possess the potential to regenerate the lost myocardium with robust *de novo* force-producing tissue.

## Conclusions: current answers to the questions on connexin expression and gap-junctional intercellular communication in ES/iPS cells

It seems reasonable to conclude that mRNAs encoding almost all of the connexins are expressed in ES/iPS cells. At protein level, however, expression of only a few connexins, such as Cx43, Cx45, Cx31, and Cx40, has been confirmed. Many studies have shown that undifferentiated ES/iPS cells communicate with each other via gap junctions at a high level. Several studies using Cx43 RNAi demonstrated that Cx43 contributes substantially to a high level of GJIC in undifferentiated ES/iPS cells.

Concerning changes in connexin expression and GJIC during differentiation of ES/iPS cells, it has been shown that expression of tissue-related connexins, such as Cx40, Cx43, Cx45, and Cx37 in the cardiomyocyte, is upregulated and that GJIC between pluripotent and differentiated cells is disrupted, resulting in formation of “communication compartments.” Regarding changes in connexin expression and GJIC during induction of pluripotency in somatic cells, the studies mentioned here have demonstrated that GJIC is re-established and Cx43 expression is upregulated during reprogramming to pluripotency.

Cx43-mediated GJIC has been shown to play an important role in maintenance of pluripotency. In fact, pharmacological blockers of GJIC and Cx43 downregulation by siRNA have been shown to induce a loss of their pluripotent state in mouse ES cells (Todorova et al., 2008). Cx43 has also been shown to play an important role in reprogramming, possibly by GJIC-independent mechanism including effects on the MET process (Sharovskaya et al., 2012; Ke et al., 2013).

Because the literature on connexin expression and GJIC in iPS cells is limited, it is difficult to conclude whether there are differences between ES and iPS cells at present. However, available data suggest that human ES and iPS cells share a similar feature concerning expression and function of connexins.

Although important roles of connexin expression and/or GJIC in ES/iPS cells can be currently perceived, many critical questions including precise mechanisms by which connexin expression influences pluripotency and reprogramming remain to be clarified.

### Conflict of interest statement

The authors declare that the research was conducted in the absence of any commercial or financial relationships that could be construed as a potential conflict of interest.
